# *In-situ* particles reorientation during magnetic hyperthermia application: Shape matters twice

**DOI:** 10.1038/srep38382

**Published:** 2016-12-06

**Authors:** Konstantinos Simeonidis, M. Puerto Morales, Marzia Marciello, Makis Angelakeris, Patricia de la Presa, Ana Lazaro-Carrillo, Andrea Tabero, Angeles Villanueva, Oksana Chubykalo-Fesenko, David Serantes

**Affiliations:** 1 Department of Physics, Aristotle University of Thessaloniki, GR-54124 Thessaloniki Greece; 2 Instituto de Ciencia de Materiales de Madrid, CSIC, Cantoblanco, ES-28049 Madrid, Spain; 3 Instituto de Magnetismo Aplicado (ADIF-UCM-CSIC), Las Rozas, Madrid 28230, Spain; 4 Departamento de Física de Materiales, Universidad Complutense de Madrid, Ciudad Universitaria, 28040 Madrid, Spain; 5 Departamento de Biología, Universidad Autónoma de Madrid, Cantoblanco, 28049 Madrid, Spain; 6 IMDEA Nanociencia, Faraday 9, Cantoblanco, Madrid, Spain; 7 Applied Physics Department and Instituto de Investigacións Tecnolóxicas, Universidade de Santiago de Compostela, 15782, Spain; 8 Department of Physics, University of York, Heslington, York YO10 5DD, United Kingdom

## Abstract

Promising advances in nanomedicine such as magnetic hyperthermia rely on a precise control of the nanoparticle performance in the cellular environment. This constitutes a huge research challenge due to difficulties for achieving a remote control within the human body. Here we report on the significant double role of the shape of ellipsoidal magnetic nanoparticles (nanorods) subjected to an external AC magnetic field: first, the heat release is increased due to the additional shape anisotropy; second, the rods dynamically reorientate in the orthogonal direction to the AC field direction. Importantly, the heating performance and the directional orientation occur in synergy and can be easily controlled by changing the AC field treatment duration, thus opening the pathway to combined hyperthermic/mechanical nanoactuators for biomedicine. Preliminary studies demonstrate the high accumulation of nanorods into HeLa cells whereas viability analysis supports their low toxicity and the absence of apoptotic or necrotic cell death after 24 or 48 h of incubation.

Magnetic nanoparticles have been intensively investigated for *in-vivo* biomedical applications within the last decades^1^, based on the harmless effects of magnetic fields to the human body and on the high biocompatibility of iron oxides. Such features motivated a plethora of research efforts in various areas, ranging from cancer diagnosis^2^ to gene therapy^3^ or magnetic resonance imaging^4^. A particularly active research area is *magnetic particle hyperthermia* (MPH), within the wide research field of the so-called *magnetic nanorobotics* (MNRBs). MPH refers to the heat release by magnetic nanoparticles when subjected to an external magnetic AC field (H_AC_) finding promising applications for cancer treatment^5^ or heat-triggered drug release^6^. MNRBs refer in a general way to the remotely controlled actuation of magnetic entities within the human body to trigger specific responses^7^ (e.g. drug delivery or enhanced resonance imaging). Efforts to improve the efficiency of nanomagnets for MPH include fine-tuning of the size^8^/anisotropy^9^ and of the interaction conditions^10^ (to optimize the dose/response ratio); efforts for achieving accurate MNRBs applications include the geometrical design of optimized structures (examples are helical structures for independent positioning^11^ or particle-aggregation for improved imaging contrast^12^). The objective of the present work is to report on the synergy between heat release and self-induced directional reorientation that controls the magnetic behaviour of magnetic nanorods under an AC field during a hyperthermia process. Such simultaneous –and complementary- hyperthermic and mechanical actuation can be achieved by specifically-designing the dimensions of the rods, thus conferring those nanosized magnets double functionality as magnetic nanorobots.

The shape of nanomagnets has been reported widely to be a way to control their properties due to the appearance of additional anisotropies^13^. For example, shape anisotropy for magnetite is of the same order of magnitude than crystal anisotropy for nanoparticles with aspect ratios of 1.1 and increases up to two orders of magnitude for axial ratios larger than 5^14^. Magnetic nanoparticles assembled into chains have been described as efficient MPH agents based on their multiplicative heating effect^15^. Importantly, this occurs regardless of their initial orientation with respect to the AC field (in a viscous environment of a weakly formed gel structure; agarose concentration below 0.5%). It is the reorientation, undergone by the chains along the AC field direction, which adds, to such elongated structures, promising potential as nanoactuators^16^ in addition to their good hyperthermia response. Enhancement of the heating efficiency has also been observed for an aqueous suspension of spheres after texturing in a DC field parallel to the AC field^17^. However, under usual conditions, where the nanoparticles are linked by weak electrostatic and/or magnetic dipolar coupling, their physical robustness during the AC application is questionable, and result in chain breaking thus reduced performance. This low sustainability of the nano-formations needs to be avoided for *in-vivo* treatments, which require a controlled and repeatable consistent response within the body. Aiming to overcome such limitations, current research efforts are mainly focused on mechanical reinforcement of the elongated structures, either bio-inspired (magnetotactic bacteria^18^); or created by design (e.g. using nanotubes as artificial building blocks for embedding the particles^19^). However, to date both approaches have shown only limited success; and both have inherent difficulties for achieving large scale together with size control, concluding in scattering of the heating output and reorientation performance. In the present work, we investigate alternative magnetic nanostructures more physically robust against both thermal and directional reorientation processes, able to display both hyperthermia-enhanced features and good magneto-mechanical performance remotely controlled by AC fields. Next we explain our criteria for designing the ideal nanomagnet fulfilling those requisites.

In general, in order to have a significant response to the AC field the nanomagnet must have a large magnetic moment. Moreover, an elongated shape increases the area of the hysteresis loop and leads to a large magneto-mechanical torque. One could think that a good candidate possessing both characteristics would be a magnetic nanowire, of very high aspect ratio and large magnetization at remanence. However, it is important to keep in mind that the magnetic nanostructure must be not only *physically*, but also *magnetically* robust: the reversal process must exclude non-coherent demagnetisation processes which lead to smaller heating capabilities (i.e. decrease of the coercivity) as well as to lower magneto-mechanical torque. Magnetic nanowires, which usually undergo non-coherent reversal mechanisms^20^, are thus eventually not suitable candidates for enhanced heating outcome. Accordingly, nanomagnets for simultaneous good heating performance and efficient physical reorientation must have the following characteristics: i) large magnetic moment, ii) large geometrical aspect ratio, and iii) reverse via coherent (or quasi-coherent) energy-dissipative paths. In order to collect some knowledge of the most suitable candidates, we have, at first, theoretically investigated the largest dimensions of magnetic nanoobjects, which reverse magnetization, via quasi-coherent processes.

The natural starting point is to consider elongated structures of axial symmetry (ellipsoids) of different dimensions -both aspect ratio (long/short axes), and volume-, and to simulate hysteresis M(H) loops to gather information about the reversal mechanism. We have used micromagnetic simulations (see Computational Details Section) to determine at which nanomagnet size the transition from coherent to non-coherent reversal is expected to occur^21^. We focus on ellipsoids of magnetite based on its suitability for biomedical applications^1^, and systematically vary its dimensions. Since for the biomedical applicability, a major prerequisite is the efficient cellular uptake, we start with the smaller dimension of the ellipsoid to be set as 50 nm^22^,^23^ (though, additional factors such as coating and shape may significantly modify the optimum size for internalization efficiency^24^,^25^). This value is much lower than the theoretical estimation for the transition from single-domain to multi-domain size for magnetite (around 80–90 nm in diameter)^26^ and hence a single-domain configuration may be expected. Note, however, that regarding energy dissipation what must be considered is the reversal mechanism: it is the type of reversal process -and not the magnetic configuration at the remanence-, what determines the amount of dissipated energy. Such distinction is illustrated by comparing the reversal processes of two different magnetic structures with single-domain configuration at remanence: a small spherical nanoparticle and a magnetic nanowire (NW). If the reversal process of the particle is via coherent rotation, the energy dissipated is proportional to the particle volume and anisotropy^27^; in the magnetic NW, however, the reversal is likely to occur via nucleation and propagation of a domain wall (DW)^20^, and in that case the energy dissipated is not proportional to the entire NW volume but only to that of the DW. For the present elongated particles, the additional anisotropy contribution due to ellipsoidal shape might promote the coherent-reversal behaviour even in larger sizes. Therefore the investigation about the reversal process depending on shape and size of the rods is necessary.

We simulated the M(H) loops at different angles (φ) between the AC field direction and the long axis of the ellipsoid, and evaluated the angular dependence of the coercive field (H_C_). Figure 1A shows three representative H_c_ *vs.* φ curves corresponding to different aspect ratios (length:width: 5:1, 2:1, 1:1), in all cases with a fixed width of 50 nm. The different tendencies followed with increasing ratio illustrate the transition from the spherical-shape particle, in which the magnetocrystalline energy (cubic and negative for magnetite) governs the magnetic response of the particle, to the shape-anisotropy dominant case at higher axial ratios of ellipsoidal shape. In the former case (spheres), the H_C_ *vs.* φ curve follows the characteristics of the double-fold symmetry (coming from the cubic anisotropy) in the 0°–90° range^28^; whereas in the latter case (ellipsoids) the H_C_ *vs.* φ curve displays the characteristic fingerprint of coherent-like reversal^21^. The change in the behaviour is due to the magnetostatic contribution to the effective anisotropy, which becomes progressively important until completely dominates^29^. It is worthy to mention here, that for large systems, higher ratios do not lead to the same gain in the H_C_ values at low angles, reflecting the transition to non-coherent reversal processes. From the detailed analysis of the particles’ magnetization during reversal (see Scheme S1 within the Supporting Information for some illustrative snapshots), we conclude that this transition occurs when varying the particle dimensions from 200/40 nm aspect ratio, to 300/60 nm. This is also confirmed by a more detailed study also varying sizes while keeping the same ratio (see Fig. 1B), or even slightly deviating from it (e.g. for the 250/60 nm ratio case). Therefore, in the first approximation the simulations predict ellipsoidal magnetite particles of 250/50 nm ratio to be the most adequate candidates for large heating and large magneto-mechanical coupling under an external AC field. These nanoparticles have the largest magnetic moment still retaining the coherent magnetization-reversal mechanism.

Under this framework, we have synthesized ellipsoidal magnetite particles of approximately similar dimensions (see Fig. 1C) using a procedure previously reported^30^. The details of the synthesis procedure can be found in the Experimental Details section. The magnetic characterization (see M(H) curves displayed in the Supporting Information, Figure S1) shows that the particles do not undergo the superparamagnetic transition at high temperature (possessing at 250 K, H_C_ = 180 Oe and M_r_ = 0.3 M_S_), thus being adequate driving vectors for deep targeting with respect to their large magnetic moment^31^. We have evaluated the hyperthermia performance of the synthesized nanorods (Fig. 2), following the same procedure as previously reported^15^. The obtained Specific Absorption Rate (SAR) values for particles dispersed in water at 5 mg/mL at low field (150 Oe) and low frequency (210 kHz), are lower than 10 W/g (see inset in Fig. 2A). However, SAR reach values as large as 759 W/g_Fe_ under an AC field of 300 Oe amplitude and 765 KHz frequency (Fig. 2A). These large values make the nanorods suitable candidates as MPH agents (for a detailed discussion on the performance of the magnetic nanorods under different frequencies see Supplementary Information).

Moreover, we observe that the ellipsoids show an enhanced heating -approximately double- than spherical particles (of diameter similar to the nanorod width) distributed at random, yet not as great as when particles assembled into chains, studied in a prior work^15^. For spheres, of equivalent volume, (85 nm in diameter), the energy loss has been reported to decrease^5^. Therefore, it can be partially concluded that elongating improves heating, but not as much as chaining. These results (Fig. 2A) correspond to rheological conditions of a low-viscous liquid with high particle mobility, whereas here, we aim to seek for robust magnetic structures at higher viscosity surroundings. Consequently, we have performed SAR measurements at fixed position samples obtained by gelating particle solutions inside a high agarose content (3% wt) matrix. For the sake of comparison, with the chains, we considered two different cases: a random distribution of the ellipsoids, and a magnetically driven assembly in elongated structures (see insets in Fig. 2B). For the latter case the SAR has been measured applying the field both parallel (0°) and perpendicular (90°) to the chain axis. The results are displayed in the Fig. 2B. At first sight, SAR values a) reach notable increasing levels when the AC-field exceeds the coercive field and b) are significantly reduced, as expected^15^, at higher viscosity conditions.

Furthermore, we also observed a large difference in SAR depending on the arrangement conditions. In spherical particles assembled into chains (prior work^15^) such difference was interpreted in terms of the enhanced effective anisotropy along the chains resulting from their AC-induced reorientation (though, in a weakly formed agarose matrix), thus enhancing the SAR along the chain direction. This process is illustrated in Fig. 3A and C, in which the angular-dependent M(H) hysteresis loops show the change from random case to the oriented one: the M(H) hysteresis curves are angle-independent in the random case, but show a marked angular-dependence in the oriented one, evolving from wide loops in the direction parallel to the AC field to narrow ones in the perpendicular one. However, this does not seem to be the present case (what would correspond to Fig. 3D), in which the enhanced heating occurs in the perpendicular direction. The origin of this difference is the strong shape anisotropy of the ellipsoids, which counterbalances the effect of chaining (even rendering it negligible). In fact, such second-order role of the interparticle coupling is supported by the moderate effect of interactions in the SAR values when the system is at random (Fig. 2A), thus pointing out the determining role of the shape anisotropy of the ellipsoids in the heating performance. In this context, the SAR-enhancement observed in the perpendicular direction would correspond to the ellipsoids oriented perpendicularly to the chains, as illustrated in Fig. 3E. Note that the perpendicular-to-the-chain arrangement of elongated particles has been observed in the past^32^ but not in the time-domain of magnetic hyperthermia. Unfortunately, the SEM images in such high-agar conditions do not provide enough resolution to check the existence of some preferential orientation of the ellipsoids. Additionally, it could also happen that the AC field induces reorientation of the ellipsoids and that needs to be taken into account as well. In order to study such reorientation issues it is necessary, therefore, to infer the spatial orientation of the chains after the AC field. For such purpose we designed the following procedure:

(i) Random dispersion of nanorods in a 3% wt agarose solution. Specimens were casted in a cylindrical shaped plastic mold and quenched to 20 °C. A particles’ concentration of 6 mg/mL was used to improve heating power and increase magnetic detectable signal; a lower particles’ concentration would not allow reaching temperatures close to the melting point of agar’s structure (~45 °C) under the used AC field conditions (300 Oe, 765 kHz).

(ii) Application of the AC field (300 Oe, 765 kHz) for sufficient time to reach and control temperature at around 46°–47 °C for 30 min in order to achieve the partial melting of agar’s structure and thus ease the possible reorientation of the ellipsoids.

(iii) Thermo-reversible gelation of the specimens after AC field treatment by quenching at 20 °C, in order to preserve the possible AC field-induced reorientation.

(iv) Measurement of angular-dependent M(H) major-loop quasistatic curves to determine hysteresis losses (HL) as the area of the loops, for inferring the spatial orientation.

In order to ensure that the DC magnetic field used during the M(H) measurements does not induce a preferential orientation in the measurements, the same angular-dependent measurements have been performed on a control sample (randomly distributed, but without applied AC magnetic field treatment). The results illustrating the angular-dependent procedure and the possible reorientation scenarios are displayed in Fig. 3 with 3A and 3C referring to spherical particle-chain samples, at random and aligned, respectively.

Figure 3B corresponds to random rod sample while 3D and 3E illustrate the two main possibilities regarding AC field induced reorientation of the nanorods: that they align along or perpendicular to the AC field direction, respectively. In order to infer which is the case occurring in the experiment, the HL values measured as a function of the angle between the experimental AC field and the DC measurement one, *η*, are plotted in Fig. 4A. The fact that the HL values increase until *η* = *90°* and then decrease again (symmetrically), indicates that the ellipsoids are oriented in the direction perpendicular to the AC field. The HL value is maximum when the ellipsoid lays parallel to the DC field, and minimum in the perpendicular direction. In order to ensure that this is always the case in ellipsoidal nanomagnets, we considered a plethora of other possibilities (e.g. different surface anisotropy, exchange values, etc.) to check whether other possible reversal mechanisms could lead to different angular dependence. The results in all cases showed similar trends (see Figure S7 within Supporting Information). We can conclude, therefore, that the AC field treatment induces perpendicular reorientation in ellipsoidal rods, offering novel perspectives for complementary use with nanoparticle chains^15^ or nanoplatelets^33^, which reorient in the parallel-to-the-field direction. Furthermore, this result for the first time confirms, to the best of our knowledge, the theoretical predictions by Usov *et al*.^34^ regarding the possibility of perpendicular reorientation during a hyperthermia treatment.

Once we have established the perpendicular reorientation of the ellipsoids, another issue that deserves further investigation is which is the attained degree of collinearity within the sample. It is likely that after the AC field application, the nanorods will not all be oriented at the same angle: different effects as interparticle coupling, irregularities in the magneto-mechanical torque or viscosity likely prevent any complete reorientation. Eventually, knowing the degree of collinearity becomes extremely crucial because; (i) the relative angle between easy axes and AC field determines the heating performance for a given field amplitude^35^, and (ii) it is directly related to the amplitude of the nanorods oscillations for its possible use as nanoactuators. In order to investigate the degree of non-collinearity, we have simulated the angular-dependent hysteresis loops for a system of non-interacting ellipsoids under different collinearity conditions. All the simulations were performed in the conditions of major hysteresis loops. The non-interacting assumption is supported by the essentially concentration-independent SAR values reported in Fig. 2A. Collinearity variation is described by the angle Ω corresponding to the cone that englobes the spatial orientations of the ellipsoids with respect to their long axis (see the cones depicted in Fig. 3D and E for the parallel and perpendicular cases, respectively). The comparison between experimental results and the angular-dependent HL values simulated at different Ω-conditions allows us to infer the spatial configuration in the real samples. We have obtained that the more similar trend between experiments and simulations corresponds to the Ω ~ 30° in the perpendicular direction, as shown in Fig. 4A. The inset shows the comparison between the parallel and perpendicular nanoparticle arrangements (see Fig. 3D and E, respectively) cases for that particular collinearity, illustrating the opposite trends. It is also worth to recall that the experimentally measured HL-values for the non-oriented case (as depicted in Fig. 3B) are essentially angular-independent, what supports our initial guess that the DC field used in the angular measurements would not produce a significant effect in the spatial orientation of the nanorods. All together these results confirm the suitability of the designed procedure to envisage the AC field induced reorientation in the sample.

From Fig. 4A it seems natural to raise the following question: is it possible to influence the degree of collinearity by the AC field characteristics? In order to check this issue we have repeated the same procedure, but changing the duration of the AC field treatment. The increase in the treatment duration allows more time for mechanical relaxation under high viscosity conditions. The maximum HL values in the perpendicular direction (ΔHL_max_, as shown in Fig. 4A), as a function of the AC field duration are displayed in Fig. 4B. An increase in HL value (which corresponds to a higher degree of collinearity) is clearly observed until saturation is reached. This result indicates that the degree of perpendicularly-induced collinearity can be easily controlled with the duration of the AC field treatment within a short time-window.

In summary, our results outline that nanorods of ellipsoidal shape display a promising double-functionality for biomedical applications: an enhanced hyperthermia heating performance when compared to spherical counterparts together with a manoeuvrable (orthogonal to the field) reorientation that can be easily manipulated via the AC field duration. Such synergy between heating and reorientation may open new opportunities for accurate nanomagnetic actuation within biological tissues. Thus, while hyperthermic- and mechanically-triggered cell damages have usually been considered as belonging to different frequency domains^36^, we show here that they may act simultaneously promoting novel multimodal treatment schemes.

Yet, one important additional issue to discuss is related to the large size of the particles and their biocompatibility and hyperthermia efficiency. On one side it is worth to mention that the size of the synthesized particles falls within the range (200–400 nm) recalled as the suitable dimensions to ease its extravasation into the tumour^37^, size that has however been discarded as hyperthermia mediators based on their poorer heating efficiency^38^,^39^. But those studies were based on spherical shape (either single-core or multicore) nanoparticles, and hence it becomes very important to check whether the elongated shape makes a difference. On the other side, elongated particle of similar dimensions have been reported to exhibit a remarkably good cell uptake, what would support their use for remote *in-vivo* nanoactuation^25^. In order to check this question, human cervical adenocarcinoma cells (HeLa line) were exposed to 0.1 mg/mL of nanorods for 24 or 48 h. Figure 5A shows representative Prussian blue staining images for cells incubated with nanoparticles. After incubation for 24 h, an efficient cellular uptake and accumulation of nanoparticles, distributed as punctate structures throughout the cytoplasm, were detected within HeLa cells. Furthermore, cells incubated for 48 h did not show a significant increase in nanorods internalization compared to 24 h of exposure, suggesting that cellular uptake of ellipsoidal magnetic nanoparticles became saturated. Similar results related to saturation of nanoparticle uptake have been described for other magnetic nanoparticles^40^ and different silica nanoparticle geometries^41^. It is important to note that the successful internalization of this nanomaterial inside HeLa cells did not induce any detectable change concerning its morphology, size and shape compared with untreated control cells. Taking into account the possible cytotoxic effects associated to magnetic nanoparticles^42^, HeLa cell viability was analysed by both MTT and Trypan blue assays. MTT results demonstrated that cell viability was not significantly affected by the presence of nanorods at 24 h of treatment (see Fig. 5B). However, a slight (survival rate 90.3%) but statistically significant increase cytotoxicity was detected after 48 h of incubation. Considering that MTT assay has been partially questioned to evaluate magnetic nanoparticles toxicity^43^, we have carried out also Trypan blue dye exclusion assay. Several articles have proposed this assay as the gold standard method to validate the cell viability after magnetic nanoparticle incubation^44^,^45^. The results obtained by Trypan blue assay (see Fig. 5C) corroborate the biocompatibility of these nanorods. Further, as shown in Fig. 5D, incubation with nanorods did not cause significant early apoptotic and late apoptotic/necrotic cell death after 24 or 48 h of incubation, evaluated by apoptotic Annexin V/PI staining assay. In summary, this iron oxide nanorods have a high degree of biocompatibility related to other ellipsoidal magnetic nanoparticles, bearing in mind that cytotoxicity has been evaluated for periods not exceeding 24 h^46^.

The goal of the present work was to study the role of particle shape for its performance under AC fields. Because of the intrinsic complexity of the simultaneous experimental measurement of the proposed objectives -heating and reorientation-, using relatively large particles (with large magnetic moment), was beneficial in order to obtain detectable signal; and using a large frequency and amplitude of the fields was also beneficial to induce significant (easy to discern) action on the particles. In this regard, an arguable aspect to consider regarding the direct *in vivo use* of our experiments is related to the field conditions (300 Oe, 765 kHz). For several years, a maximum field-frequency product *H* × *f* ≤ 4.85 × 10^8^ A m^−1^s^−1^ (the Atkinson-Brezovich limit^47^) was considered as the acceptable threshold for the safe clinical use, to avoid resistive heating in the biological tissues. The conditions used in our experiments exceed that value for up to 2 orders of magnitude (*H* × *f* = 2.5 × 10^9^ and 18.3 × 10^9^ A m^−1^s^−1^ for the 150 Oe, 210 kHz; and 300 Oe, 765 kHz experiments, respectively), what might be considered as inadequate for *in vivo* treatment. However, exhaustive experiments and analysis performed have shown that in all the considered cases the allowable values of *H* × *f* are much larger than the Atkinson-Brezovich limit, ranging between 1.8 × 10^9^ A m^−1^s^−1^ (B. Thiesen & A. Jordan; 1st clinical magnetic hyperthermia application^48^); to 8.3 × 10^9^ A m^−1^s^−1^ (S. Kossatz *et al*.^49^) and up to 18.7 × 10^9^ A m^−1^s^−1^ (H. Mamiya^50^). It should be mentioned here that despite the use of a relatively high frequency (765 kHz), analogous frequencies have been utilized in *in vitro* studies to overcome the limited heating efficiency^51^. Specifically, in applications where nanoparticles are involved, nanoscale tissue regions may tolerate larger field-frequency products if we account for additional parameters that affect nanoscale tissue heating^52^. Additionally, we have examined our experimental sequence (765 kHz, 27.4 kA m^−1^) *in vitro* and found that is very well penetrated by normal and cancer cell lines (C2-C12, 3T3-L1 normal cells and MDA-MB-231, SkBr3, MCF7 breast cancer, SaOS-2 human osteosarcoma cells: *See control samples without MNPs*)^53^,^54^,^55^.

Finally it is also worth to point out that throughout the entire work, despite not mentioning it explicitly, we have implicitly interpreted our results considering Néel rotation as the heating source. Such interpretation is borne out by several works^56^,^57^ - including our own experiments^58^- reporting a small (or even inexistent) contribution of physical rotation to heat dissipation. With the present work we have demonstrated that even if the physical rotation contribution is not a heating source *per se*, it may indirectly play a crucial role leading to a re-orientation and thus influencing the overall heat output. To sum up, ellipsoidal magnetic nanoparticles have properties that make them suitable for future cancer treatment by magnetic hyperthermia, including their magneto-mechanical response to an external AC magnetic field, tumour cell labelling efficiency and biocompatibility.

## Materials and Methods

### Computational details

We used the OOMMF software package^59^ to simulate the hysteresis properties of the rods of different sizes. The material characteristics are those of the magnetite (cubic anisotropy, K = −1.1 * 10^5^ erg/cm^3^; M_S_ = 477 emu/cm^3^; exchange stiffness A_exch_ = 1.0 × 10^−6^ erg/cm). We checked different initial configurations for the ellipsoids (vortex, random, or saturated state), and no difference was obtained in the simulated magnetization processes. The surface anisotropy was simulated as pointing perpendicularly to each point, and assumed to influence the outer 5% cubic cells.

### Synthesis and surface modification of magnetite ellipsoidal nanoparticles

First, goethite was synthesized by precipitation of Fe(II) sulphate with sodium carbonate and the resulting dispersions were oxidized at constant temperature (40 °C) by bubbling air at a constant flow following the method described in a previous work^60^. The goethite particles were first heated for 4 h in air for dehydroxylation, then coated with silica with TEOS and reduced, at constant temperature 360 °C in a hydrogen atmosphere for 3.5 h.

### Preparation and orientation of agarose specimens

For randomly oriented specimens, nanoparticles were dispersed in a hot agar’s solution (3% wt) at 70 °C under sonication and then cooled down to 20 °C. For the orientation of nanoparticles in agarose matrix two approaches were followed: (i) pre-orientation by subjecting particles dispersions in a static magnetic field during cooling and (ii) AC-field orientation of randomly-oriented specimens by the partial melting during induction heating under the AC field followed by cooling. In the second case, the temperature of the sample was allowed to rise above 45 °C and then controlled in the 46–47 °C range by switching on/off the field.

### Hyperthermia and angular-dependent measurements

The heating efficiency of nanoparticles dispersions in water and agarose matrix was measured under a low (210 kHz) and high frequency (765 kHz) AC magnetic field at intensities ranging 150–300 Oe. A 1.2 kW Ambrell Easyheat 0112 system and a commercial converted 4.5 kW inductive heater were used, respectively. The specific absorption rate (SAR) was derived from the slope of the temperature versus time curve after subtracting water background signal and heat losses to the environment. Temperature was monitored by using a GaAs-based fiber optic probe.

Magnetic measurements of the randomly and magnetically oriented specimens were performed in a 1.2H/CF/HT Oxford Instruments Vibrating Sample Magnetometer (VSM) at room temperature. Hysteresis areas of measured loops were determined as an estimation of occurring hysteresis losses during hyperthermia treatment. For angular varying measurements, hysteresis losses were quantified as an indication of particles orientation degree.

### Cell cultures

Human cervical carcinoma cell (HeLa) line was obtained from American Type Culture Collection (ATCC^®^ CCL2™). HeLa cells were grown as monolayers in Dulbecco’s modified Eagles’s medium (DMEM) with 50 units ml^−1^ penicillin, 50 μg ml^−1^ streptomycin, and supplemented with foetal bovine serum (FBS), at a final concentration of 10%. All media, sera, and antibiotics were provided by Gibco (Paisley, UK). Cell cultures were performed in a 5% CO_2_ atmosphere at 37 °C and maintained in an incubator. Treatments were initiated three days after plating (approximately 70% confluence). Depending on the experiments, cells were seeded on 10 mm square glass coverslips placed into the wells. In order to analyze internalization of nanorods, HeLa cells grown on coverslips in 24-well plates were incubated with nanorods at 0.1 mg/mL in culture medium for 24 or 48 h. At the end of the incubation, the medium was removed by aspiration and cells washed three times with PBS; labeling efficiency was determined by conventional Prussian blue staining for iron detection according to our previously described method^24^. Images were captured by an Olympus BX61 epifluorescence microscope equipped with an Olympus DP50 digital camera (Olympus, Center Valley, PA, USA), and processed using the Adobe Photoshop 7.0 software (Adobe Systems). Biocompatibility of nanorods was evaluated by colorimetric MTT test and Trypan blue exclusion assay performed at 24 h of cells incubation (24 or 48 h) as we have described previously^24^. Furthermore, apoptotic and necrotic cell death was analyzed by Annexin V/Propidium Iodide (PI) double staining analysis by flow cytometry. This assay allowed us to detect one of the earliest events in apoptosis, externalization of phosphatidylserine (PPS) in living cells. This assay uses fluorescein-labeled Annexin-V (Annexin-fluorescein isothiocyanate (FITC)), which has a strong and specific affinity for PPS, to monitor PPS translocation that occurs because of apoptosis. Use of Annexin-V-FITC in combination with PI allows discrimination between viable cells (Annexin−, PI−), early (Annexin+, PI−) or late apoptotic cells (Annexin+, PI+) and necrotic cells (Annexin−, PI+).

For this purpose, HeLa cells 24 or 48 h after nanorods incubation were trypsinized, centrifuged for 4 min at 1200 r.p.m., washed twice in cold PBS and resuspended in cold (1X) binding buffer (Beckman-Coulter Inc., Fullerton, CA, USA) to a concentration of 1 × 10^6^ cells/ml. Then, 10 μl of Annexin V-FITC (Southern Biotech, Birmingham, AL, USA) was added to 100 μl of cell suspension; each tube was gently vortexed and incubated for 15 min on ice, protected from light. Without washing, 380 μl of cold (1X) binding buffer + 10 μl of PI (50 μg/ml; Beckman-Coulter Inc.) were added to each tube and samples were analyzed immediately.

Measurements were performed using a Cytomics™ FC500 flow cytometer (Beckman-Coulter Inc.) with an argon laser line at 488 nm and complemented with a 525 nm band pass and a 620 nm short pass filters. Statistical analysis was perform using GraphPad Prism software (GraphPad Software, Inc. La Jolla, CA, USA) and one-way ANOVA with pairwise comparisons between means.

## Additional Information

**How to cite this article**: Simeonidis, K. *et al*. *In-situ* particles reorientation during magnetic hyperthermia application: Shape matters twice. *Sci. Rep.* **6**, 38382; doi: 10.1038/srep38382 (2016).

**Publisher's note:** Springer Nature remains neutral with regard to jurisdictional claims in published maps and institutional affiliations.

## Supplementary Material

Supplementary Information

## Figures and Tables

**Figure 1:**
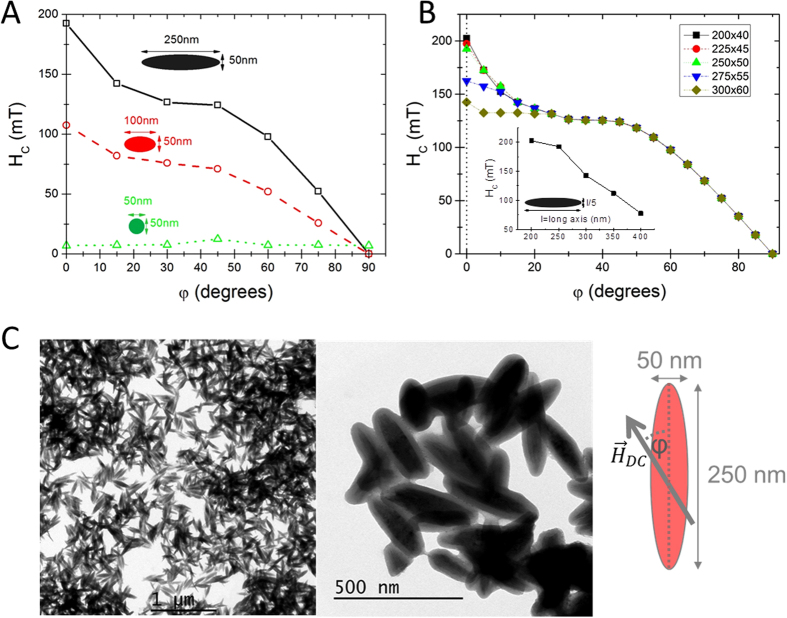
Dependence of the coercive field H_C_ on the angle between H_AC_ and the long axis of the ellipsoid, for different aspect ratios (**A**), and for different sizes for the same aspect ratio 5:1 (**B**). TEM images of the synthesized rods of ellipsoidal shape of average dimensions 250/50 nm long/short axis are shown in (**C**). The angle φ stands for the relative direction between long axis of the nanorods and the applied DC field (see Figs 3 and 4).

**Figure 2:**
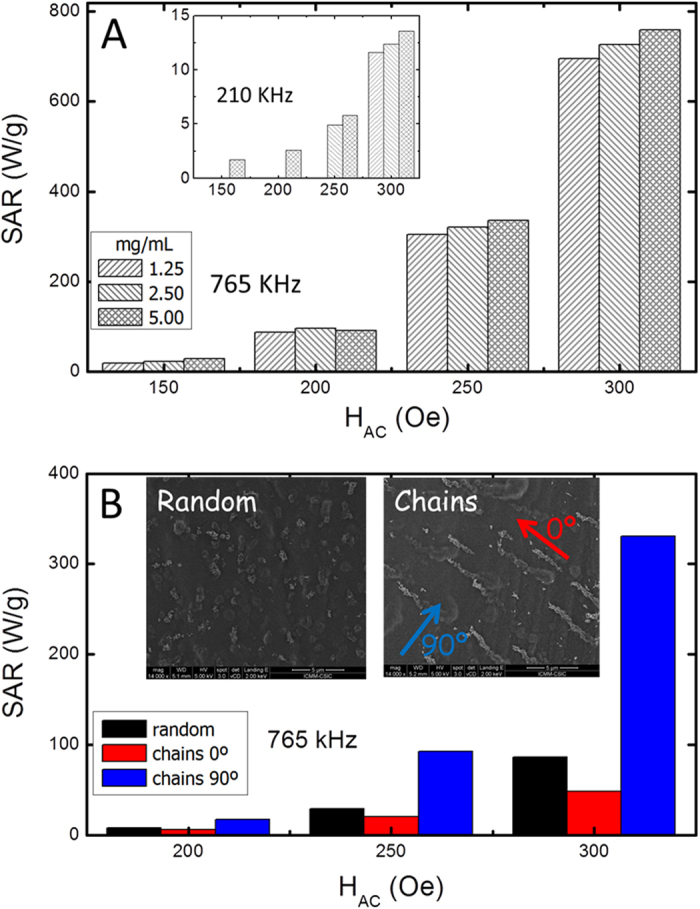
(**A**) SAR values of particles dispersions in water as a function of the field amplitude and for different concentrations (1.25, 2.50, and 5.00 mg/mL), measured at 765 kHz; the inset shows the corresponding data measured at 210 kHz. (**B**) SAR values as a function of the field amplitude, for a randomly distributed system and for a chain-like assembly in agarose matrix (3% wt); for the latter the SAR has been measured parallel (φ = 0°) and perpendicular (φ = 90°) to the chains. The insets correspond to SEM images.

**Figure 3:**
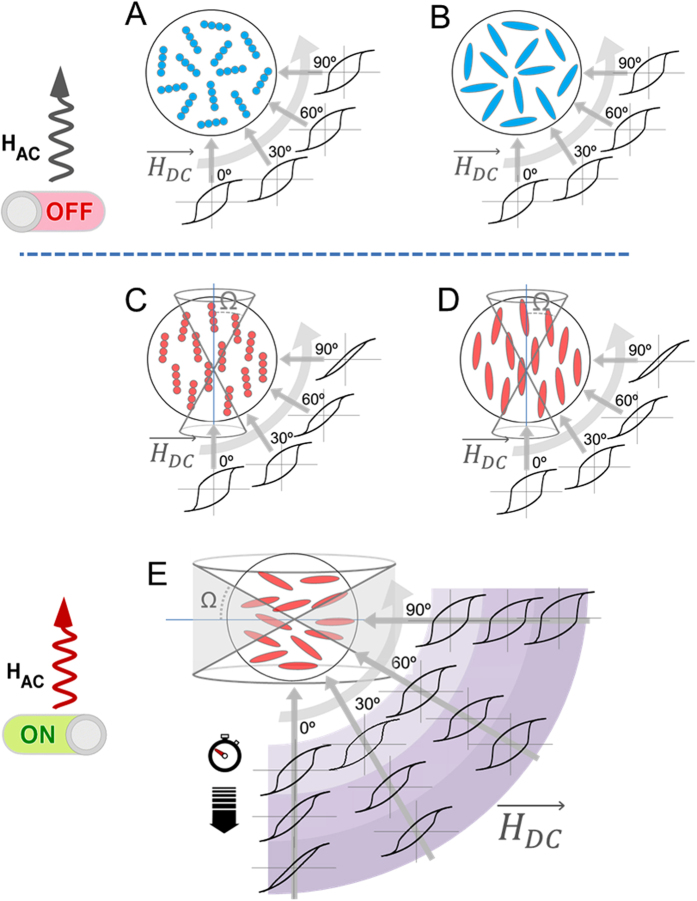
Schemes illustrating the possible effect of the AC field on the spatial reorientation of the nanorods, in comparison with the case of chains of spherical particles. Thus (**A** and **B**) correspond to the case when both systems are distributed at random (see that the M(H) hysteresis loops are angle-independent); (**C** and **D**) illustrate the reorientation of both chains and nanorods along the field, while (**E**) corresponds to the perpendicular reorientation of the nanorods. Also the effect of AC-treatment duration is illustrated in this case, with a progressive narrowing of the M(H) loops in the parallel direction with larger time. The cones stand for the collinearity-characterization angle Ω that it is defined with respect to the long axis of the nanorods in either case (parallel and orthogonal).

**Figure 4:**
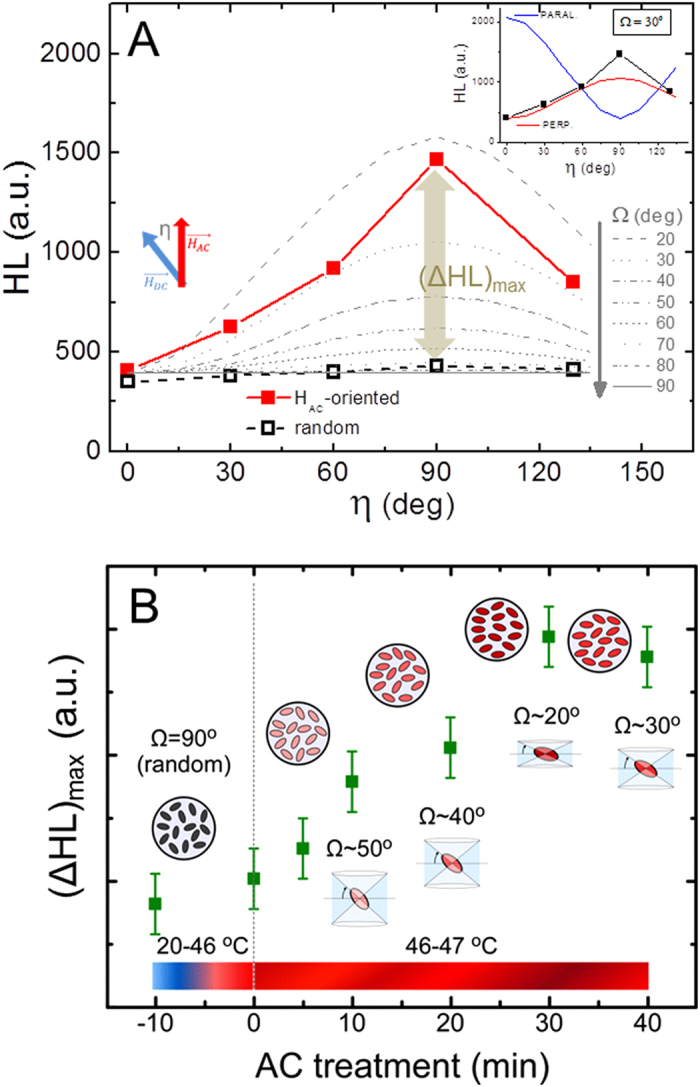
(**A**) Shows the experimentally evaluated HL values (full squares) as a function of the relative angle between AC field treatment, and DC measurement directions. The empty squares correspond to the non-oriented sample. The grey lines are the simulated angular-dependent cases for different Ω values, for the perpendicular-reorientation case (Fig. 3E). The inset shows the comparison with the parallel case, which clearly is not the experimental observation). (**B**) evolution of the HL losses along the perpendicular direction as a function of the duration of the AC treatment, which results in a different collinearity (as indicated by the corresponding Ω values).

**Figure 5:**
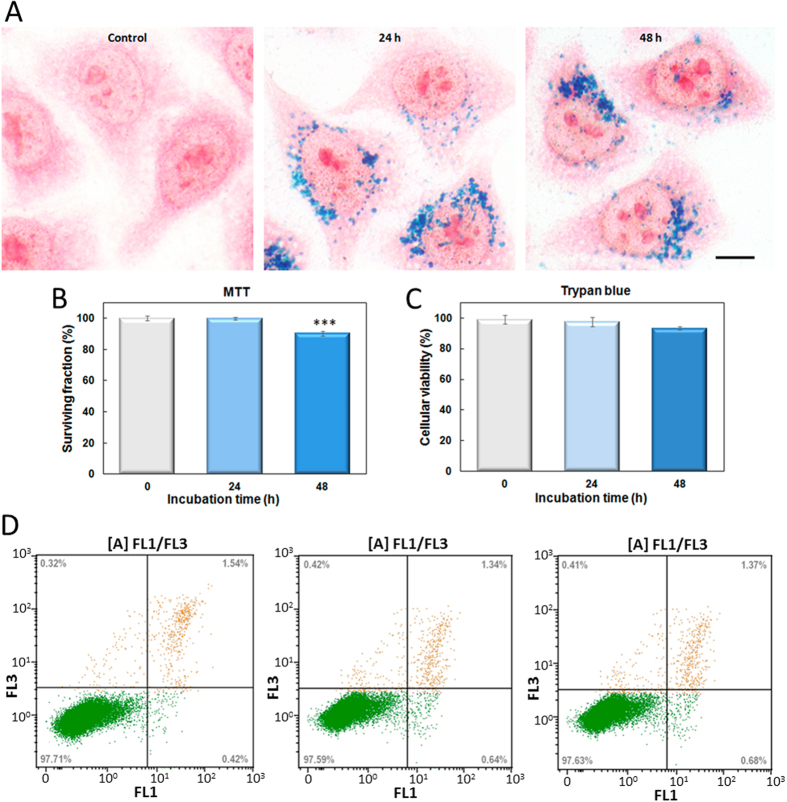
Cellular studies. (**A**) Prussian blue staining in control HeLa cells (a) or incubated with the nanorods at 0.1 mg/ml for 24 or 48 h. Scale bar = 5 μm. (**B**) MTT cell viability assay carried out after 24 h after incubation, show that the nanorods do not induced cytotoxicity on HeLa cells, but 48 h is at the limit of toxicity. Data correspond to mean ± SD values from at least six different experiments. Statistically significant differences are labeled as “***” when *P* < 0.001, for comparisons between groups using one-way ANOVA with pairwise comparisons between means (all groups versus control). (**C**) Trypan blue assay demonstrated no significant increase in dead cells number after both incubation times. (**D**) Representative flow cytometry histograms of Annexin V-FITC (FL1) in combination with PI (FL3) staining in control cells (a) or incubated with the nanorods for 24 h (b) or 48 h (c). Numbers in each quadrant indicate the percentage of cells. Similar percentages of viable, apoptotic (early and late) and necrotic cells were obtained on samples non-incubated (control) and after nanorods incubation.
