# Supplementary material for: In-situ particles reorientation during magnetic hyperthermia application: Shape matters twice
Source: Sci Rep. 2016 Dec 6;6:38382. doi: 10.1038/srep38382 (PMC5138615; doi:10.1038/srep38382)
Supplement: Supplementary Information [file srep38382-s1.pdf]

In-situ particles reorientation during magnetic hyperthermia application: Shape matters twice

*Konstantinos Simeonidis[1], Puerto Morales[2], Marzia Marciello[2], Makis Angelakeris[1], Patricia de la Presa[3,4], Ana Lazaro-Carrillo[5], Andrea Tabero[5], Angeles Villanueva[5,6], Oksana Chubykalo-Fesenko[2], and David Serantes[7,8]**

[1]Dept. of Physics, Aristotle University of Thessaloniki, GR-54124 Thessaloniki Greece

[2]ICMM-CSIC, Cantoblanco, ES-28049 Madrid, Spain

[3]IMA (ADIF-UCM-CSIC), Las Rozas, Madrid 28230, Spain

4Dept. Física de Materiales, Universidad Complutense de Madrid, Ciudad Universitaria, 28040 Madrid, Spain

[5]Dep. Biología, Universidad Autónoma de Madrid, Cantoblanco, 28049 Madrid, Spain

[6]IMDEA Nanociencia, Faraday 9, Cantoblanco, Madrid, Spain

[7]Appl. Phys. Dept. and IIT, Universidade de Santiago de Compostela, 15782, Spain

[8]Department of Physics, University of York, Heslington, York YO10 5DD, United Kingdom

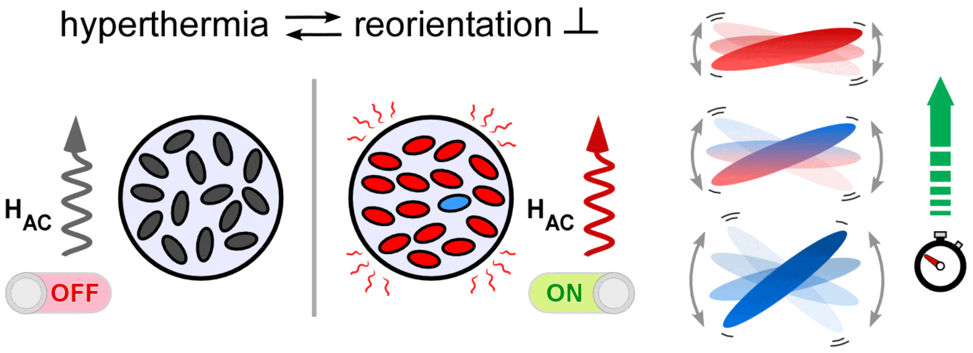

Figure S0. Sketch illustrating the time-dependent perpendicular-reorientation process induced by the hyperthermia AC field on the magnetic rods.

SUPPORTING INFORMATION

MAGNETIZATION M(H) LOOPS AND ZFC/FC CURVES

For the magnetic characterization of the samples, we measure both M(H) and M(T) curves. The measurements have been done in 50 µL of colloidal suspension at 5 mgFe /ml. The hysteresis loops have been measured at 5 T maximum applied fields. At room temperature random oriented colloid has been freeze to 5 K and to 250 K in order measure the M(H) cycles in a solid ice matrix (Figure S1). Similarly, ZFC-FC procedure has been performed at 20 mT from 5 to 260 K in order to avoid any physical reorientation of the magnetic nanorods above water melting temperature (Figure S2).

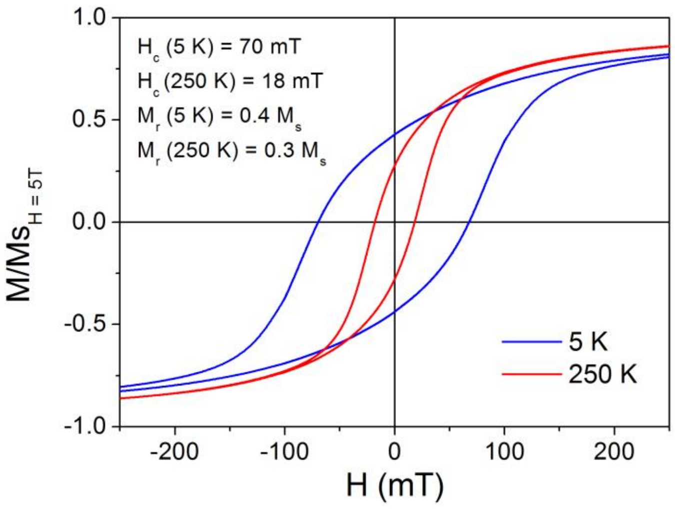

Figure S1. Hysteresis loops measured at 5 K (blue line) and 250 K (red line). The magnetization is normalized to the values at 5 T.

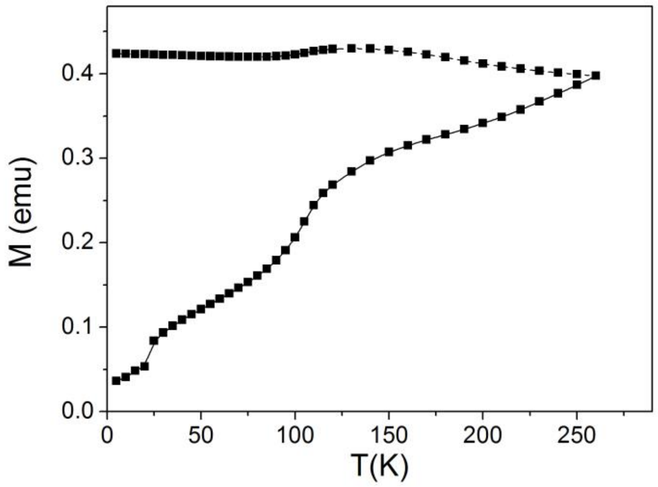

Figure S2. Thermal dependence of the magnetization measured at 20 mT from 5 to 260 K.

ANALYSIS OF THE HEATING PERFORMANCE UNDER DIFFERENT FREQUENCIES

Besides the main findings of our work, there are additional aspects in the performance of the magnetic nanorods that deserve discussion. First, it must be noted that these elongated particles experience a huge nonlinear dependence in the SAR values with varying frequency, as can be seen by comparing the SAR values in water at f=765 kHz and at 210 kHz (Figure 2A and inset, respectively). This behaviour has been also reported in other systems with spherical particles,[1] being attributed the origin of such behaviour to Brownian reorientation of the particles. This would change the relative alignment between particles' easy axes and AC field, greatly influencing the heating performance. However, in view of the relative differences in the angular-dependent measurements this does not seem to be the current case. This nonlinear *f*-dependence of the SAR clearly deserves a detailed investigation, since it strongly changes the heating performance. As a general comment we recall that the only characteristic in common is that the effect is observed in large magnetite particles, but different shapes. Thus, based on the fact that magnetite has cubic anisotropy and that in large particles magnetocrystalline anisotropy is expected to have a significant contribution, we speculate it might be related to the difference between reaching a complete magnetization reversal, or just jumping between the two adjacent energy minima (in which case the energy barrier is one order of magnitude smaller[2]). Note, nevertheless, that in this case this would stand more for the dependence on the AC field amplitude, and not just frequency. Therefore other effects as thermally-triggered reorientation facilitating a complete reversal should be taken into account; but this issue lies out of the scope of the present work.

COMPUTATIONAL DETAILS – OOMMF SIMULATIONS

The discretization size in all cases was taken as 1 nm-side cubic cell; although larger sizes up to 2.5 nm cubic side showed the same results in many cases, we decided to always use to smaller size in order to allow any possible nucleation process to occur. The results are essentially the same using an even number of cells (as in the 250/50 nm ellipsoid), than using an odd number (checked for the 251/51 nm aspect ratio ellipsoid). This was checked to ensure that no artificial results in the reversal mechanism could be originated by the chosen sizes. No thermal noise was included in order to save computational time, though additional tests including thermal fields showed the same trends.

REVERSAL MECHANISM AS A FUNCTION OF PARTICLE DIMENSIONS

To ensure the maximum reorientation efficiency, the magnetization inside the ellipsoid should be magnetically rigid. The micromagnetic analysis of reversal process for particles of different size/shape indicates the non-coherent reversal process in which vortex-like configuration appears is characteristic for nanoparticles of large sizes. This is illustrated in Scheme S1.

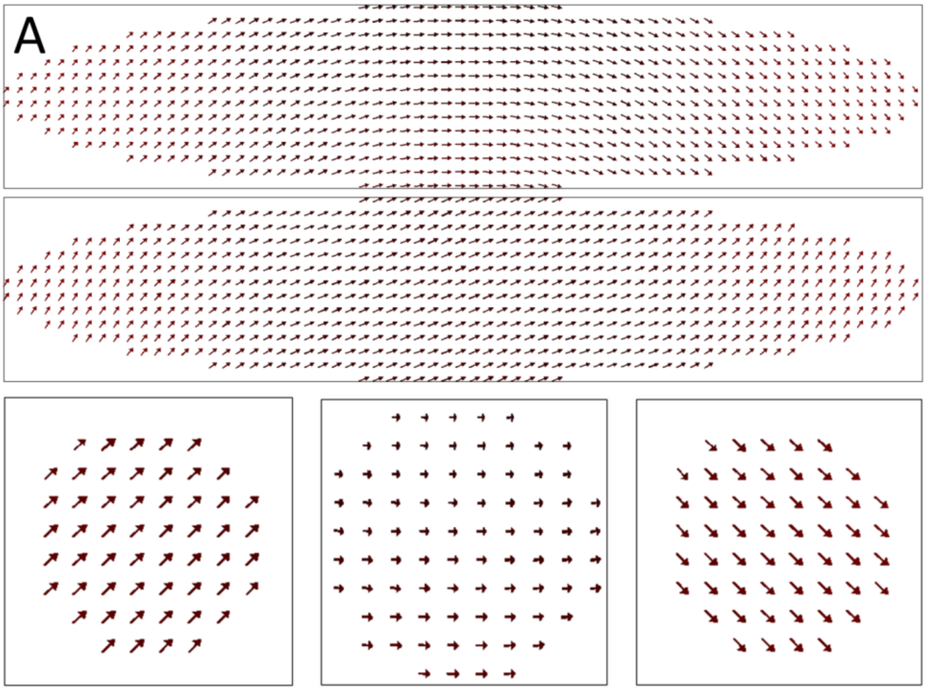

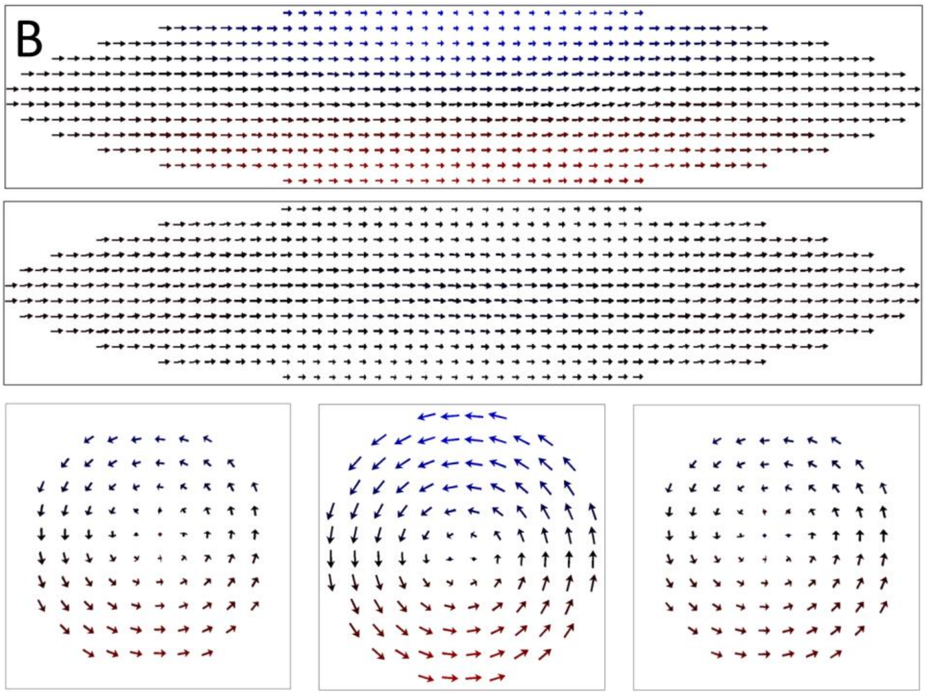

Scheme S1. Snapshots of quasi-coherent (A) and completely non-coherent (B) reversal in magnetite rods of ellipsoidal shape, of dimensions 200nm/40nm and 300nm/60nm long/short axes, respectively.

OTHER POSSIBLE REVERSAL MECHANISMS IN THE PARTICLES

In order to ensure the validity of our approach to indirectly obtain information about the spatial arrangement of the particles via their heat dissipation, we needed to confirm that effectively the highest hysteresis area corresponds to the longitudinal axis of the particle being parallel to the external field (see e.g. Figure 1 within main text). Other reversal mechanism could, in principle, lead to different cases in such large dimensions (e.g. when the shape anisotropy dominates over the magnetocrystalline one). Because of that we decided to check the role of the surface anisotropy (K_{surf}), which has been reported several times to be able to help the magnetization reversal. Different surface anisotropy may also change the functional form of the angular dependence of the coercivity. The problem to include K_{surf} is that it is expected to be present but its magnitude (or even the precise direction) is not known. We have at first assumed the following:

- K_{surf} is perpendicular to each point of the ellipsoid's surface
- there are more cells with surface anisotropy at the edges of the ellipsoid, as illustrated in the following Scheme S2 (we assumed it is present in the outer 5% of the cells in all directions):

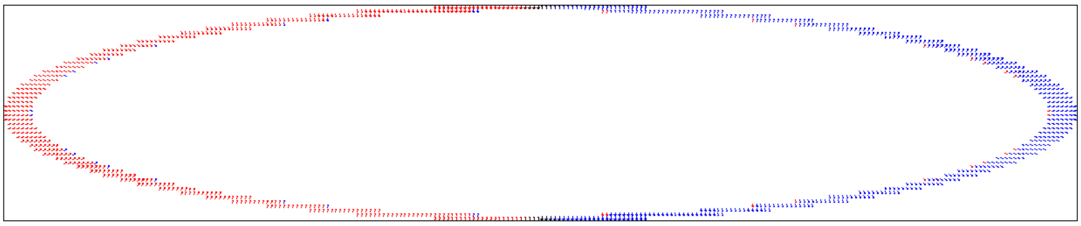

Scheme S2. Illustration of the fraction of magnetic cells in the system having surface anisotropy.

The values of the surface anisotropy were chosen so that the simulated coercive field still reasonably remains close to the experimentally measured values. Examples of hysteresis curves considering different K_{surf} values in 250nm/50nm rod are shown in Figure S4. In Figure S5 we present the evolution of $H_C(0°)$ *vs.* K_{surf} for both 250nm/50nm and 250nm/60nm rods.

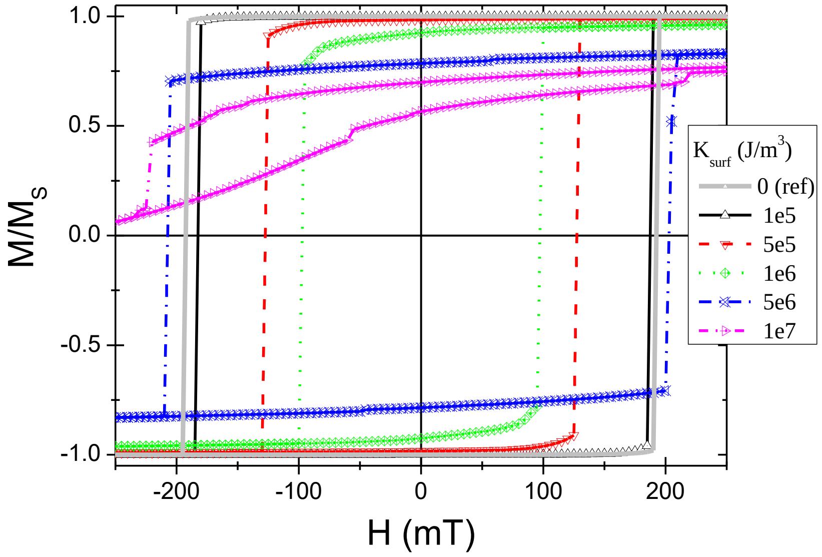

Figure S3. Simulated M(H) loops for different values of surface anisotropy in the 250nm/50nm rods.

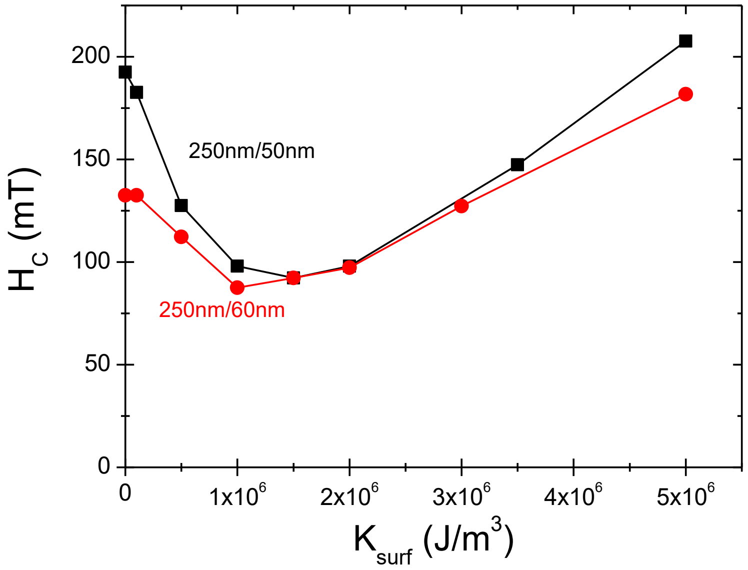

Figure S4. HC vs. Ksurf, when the field is applied parallel to the long axis of two ellipsoids of dimensions 250nm/50nm and 250nm/60nm long/short axes, respectively.

The above results indicate that surface anisotropy may either increase of decrease the hysteresis losses, and there is a given value for which increasing K_{surf}, changes the shape of the curves –even with similar H_C-, as shown in Figure S4. However, such detailed evolution is not the aim of the current investigation, in which we just need to check which is the angular dependence of the curves with or without K_{surf}. Regarding such, in Figure S5, the angular-dependent hysteresis curves of the 250nm/50nm including a surface anisotropy of value K_s=1*10^6 J/m^3 are shown.

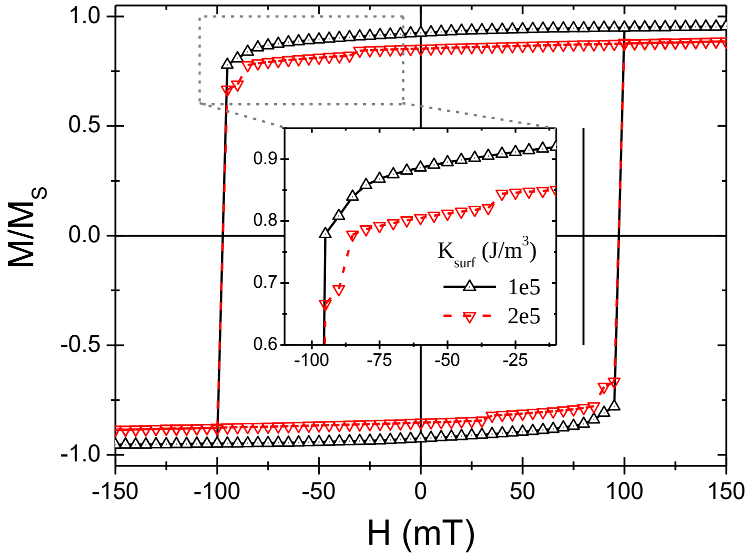

Figure S5. Change of the shape of the curves at the transition between the decrease and increase tendency (change in K_{surf} with same H_C), corresponding to the 250nm/50nm case.

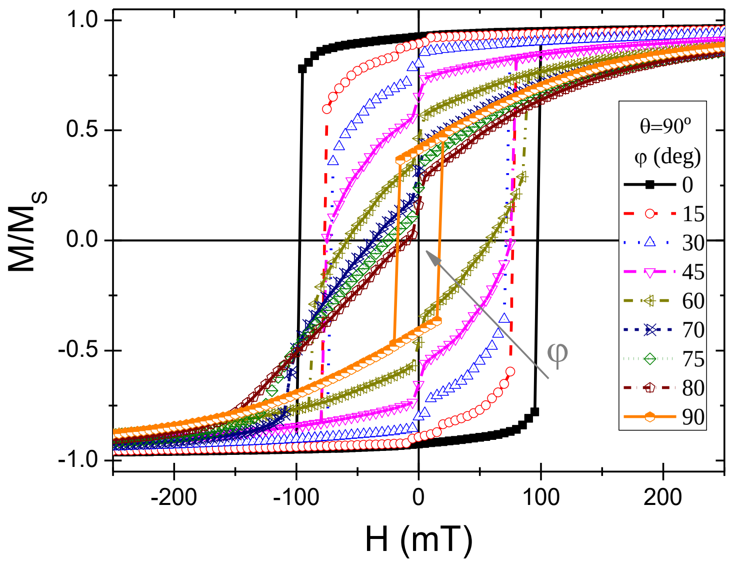

Figure S6. M(H) hysteresis loops of the 250nm/50nm case with $K_s=1*10^6$ J/m^3.

In Figure 6 it is illustrated that the maximum losses correspond to the parallel case, thus supporting our interpretation regarding reorientation direction. However, it must be also observed that for some angles the curves become anhysteretic; this aspect deserves further investigation in future works, since it could be related to the difference in the specific shape of the HL vs. angle curves in Figure 3 (main text). Finally, it is worth to note that other possibilities (different exchange at the surface; modifications in their relative values; different combinations K_{surf} vs. exchange, etc) always predict a maximum HL along the long axes of the ellipsoids. The differences are more related to the type of reversal process (e.g. change from the vortex –curling-type-, to other non-coherent but non-curling), and may influence the absolute HL value, but not significantly the angular dependence. These results are not shown for the sake of simplicity.

In Figure S6 it is observed that having surface anisotropy clearly influences the H_C value (the reduction is not large in comparison with the K_S = 0 case shown in Figure 1A within the main text), but the coherent-like reversal trend does not change. This supports our conclusions regarding the angular-dependence of HL values for inferring spatial orientation. Similar conclusions can be made considering the different A_{exch} values, as seen in Figure S7.

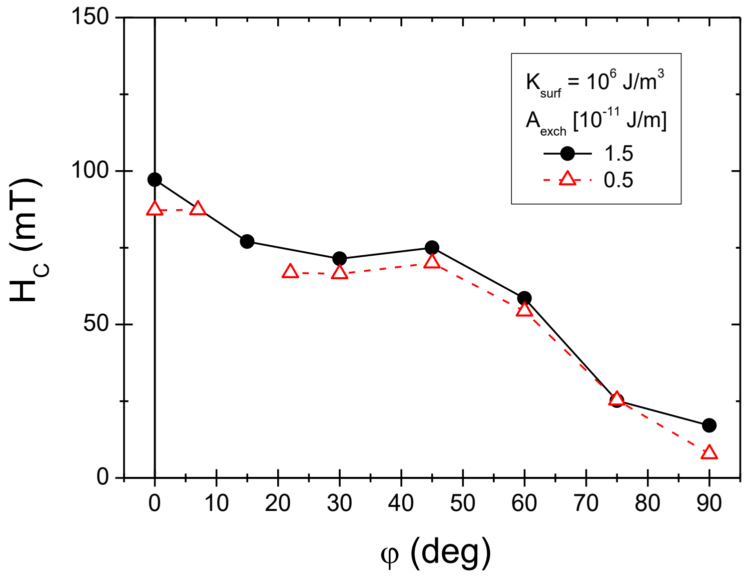

Figure S7. Angular dependence of coercivity for different values of A_{exch}., for the case $K_{surf} = 10^6$ J/m^3 that corresponds to the extremal case in Figure S5.

ESTIMATION OF THE DEGREE OF INDUCED COLLINEARITY

Based on the simulated angular dependent M(H) loops of a single-particle, we calculate the response of a random distribution of such entities (no interaction conditions), with some degree of collinearity in their spatial orientation. The average magnetization of a system of a collection of non-interacting particles along the field direction is given by

$$\langle m \rangle = \sum_{i=1}^{c} n_i(\theta_i) \left[\frac{m(\theta_i) + m(\theta_i + \Delta\theta_i)}{2} \right]$$

where $n_i(\theta_i)$ is the amount of particles between θ_i and $\theta_i + \Delta\theta_i$, given by

$$n_i(\theta_i) = \frac{1}{2\pi} \int_{\theta_i}^{\theta_i + \Delta\theta_i} sin\theta d\theta$$

Now we restrict the possible axes orientation within some cone angle (Ω) and apply the magnetic field along some direction (η). The average magnetization of the system at each angle η of the DC field with respect to the AC one, for a given Ω value, is given by

$$m_\eta = \int_0^{\Omega}\int_0^{2\pi} m(\xi)\sin\theta \, d\theta d\varphi \qquad (1)$$

where ξ=acos[sin(θ)sin(η)cos(φ)+cos(θ)cos(η)]. Note that the above expression requires redefinition of the angles depending on parallel or perpendicular reorientation estimation (regarding parallel- or perpendicular-induced reorientation, as illustrated in Figure 3D,E in the main text). To test the the code, we have generated the m(h) data (in dimensionless units of $m=M/M_S$ and H/H_A) on the Stoner-Wohlfarth model of a random system of single-domain magnetic nanoparticles with coherent rotation and uniaxial anisotropy which give a known result ($H_C/H_A=0.48$; $M_R/M_S=0.50$) for testing.

Then, we can build the averaged m(h) data at different cone-field orientations, considering both different apertures of the cone and different angles between the field and the cone, as illustrated in Scheme S3:

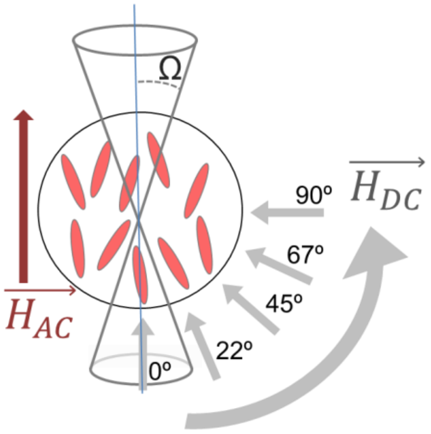

Scheme S3. Diagram illustrating the angular-dependence of the magnetization of the system on the applied field direction, as a function of the dispersion on easy axes of the particles (characterized by the cone angle Ω).

In the figure above the collinearity direction –generating the cone- points along the AC field direction under the assumption that the AC field induces this collinearity. The m(h) data corresponding to different angles between DC field and cone is plotted next, in Figure S8 (note that for the completely random case, i.e. Ω=90°, the m(h) data for the AC field applied at different angles should overlap to correspond to a truly random

system). For the present proof-of-concept case we consider the values Ω = 5°, 20°, 45°, 75°, and 90°; and the m(h) data is estimated at intervals of 15° from 0° up to 90°. The results for these examples are shown next, in Figure S8:

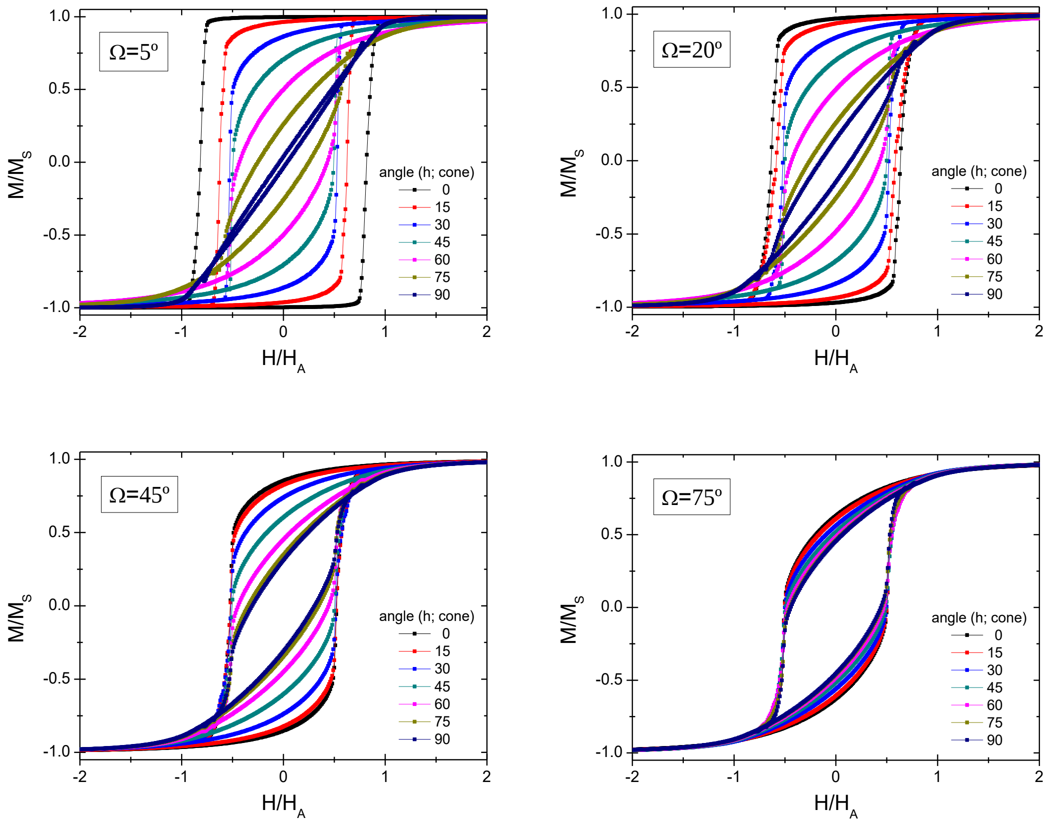

Figure S8. Angular dependence of the hysteresis loops for different dispersions of easy axes (Ω = 5°, 20°, 45°, 75°, and 90º).

The above Figure S8 clearly illustrates the expected behaviour: the more collinear the easy axes are, the more square-shaped are the loops in the parallel direction, and correspondently we have less hysteresis area along the perpendicular direction. Note that the results should be essentially angle-independent for a cone of 90°, as shown in Figure S9.

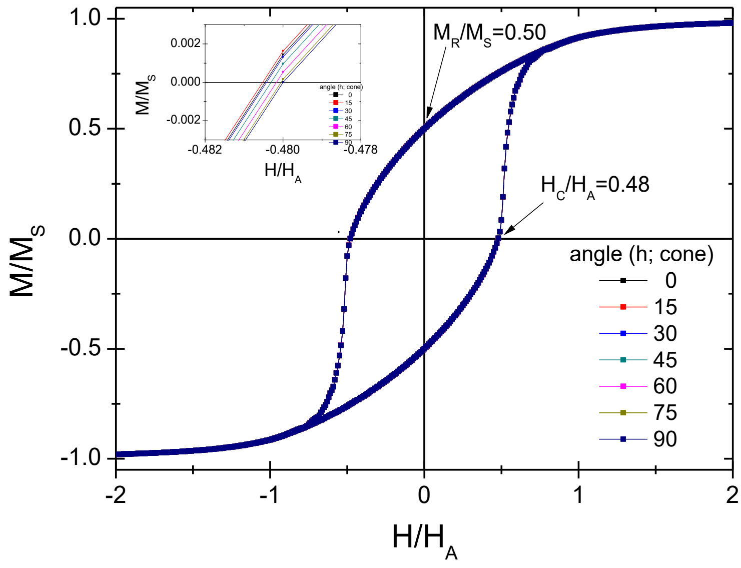

Figure S9. Angular dependence of the hysteresis loops for the random assembly (Ω = 90°).

Finally, it is worth to note that by doing so it is predicted that in the perpendicular direction there would be no HL, whereas in the experiments there is a minimum HL corresponding to the random case. We interpret this as arising from the fact that in the experiment the difference comes from a progressive increase of the amount of particles aligned in a given direction, whereas in the simulations we use always the same normalization for the population. In order to account for this effect, we have always renormalized the HL values with regard to the random case, while keeping the relative difference.

---

1. Simeonidis, K. et al. Fe-based nanoparticles as tunable magnetic particle hyperthermia agents. *J. Appl. Phys.* **114**, 103904 (2013).

2. Yanes, R. et al. Effective anisotropies and energy barriers of magnetic nanoparticles with Néel surface anisotropy *Phys. Rev. B* **76**, 064416 (2007).
